# Spatiotemporal trends of *Escherichia coli* levels and their influences vary among ponds in the coastal plain of Georgia

**DOI:** 10.1002/jeq2.70018

**Published:** 2025-03-31

**Authors:** J. Andrew Widmer, Matthew Stocker, Jaclyn E. Smith, Alisa Coffin, Oliva Pisani, Timothy Strickland, Manan Sharma, Yakov Pachepsky, Laurel L. Dunn

**Affiliations:** ^1^ Department of Food Science and Technology University of Georgia Athens Georgia USA; ^2^ USDA‐ARS Environmental Microbial and Food Safety Laboratory Beltsville Maryland USA; ^3^ USDA‐ARS Southeast Watershed Research Laboratory Tifton Georgia USA

## Abstract

Quantification of *Escherichia coli* in water is commonly used to understand a surface source's suitability for produce irrigation. Location, season, and physicochemical water quality impact the levels of *E. coli* in irrigation ponds. Water samples were collected periodically at three ponds in Southeast Georgia along a sampling grid from July 2021 through September 2023 and quantified for *E. coli* with simultaneous collection of relevant water physicochemical parameters. Mean relative differences (MRDs) were calculated for each collection point to determine differences in *E. coli* levels across sampling locations. *E. coli* levels varied significantly across sampling area (perimeter, surface, and subsurface) at each pond. The log most probable number *E. coli* 100 mL^−1^ (EC MRD) values ranged from −0.25 to 0.33 in Pond 1, −1.5 to 0.65 in Pond 2, and −1.25 to 0.65 in Pond 3. In Pond 1, EC MRD correlated positively with chlorophyll and turbidity, and negatively with dissolved organic matter, dissolved oxygen (DO), specific conductance, and pH MRDs. In Pond 2, the MRD of *E. coli* correlated with the MRDs of chlorophyll, DO, phycocyanin, pH, and temperature. In Pond 3, *E. coli* MRD correlated positively with nitrate MRD. This work showed MRD analysis may reveal stable patterns of *E. coli* and the physicochemical factors that impact these levels in ponds, though no universal covariates were identified that could estimate *E. coli* levels. These findings may provide context for water quality managers wishing to augment measurements of *E. coli* with other factors, or better represent variable *E. coli* levels with MRD.

## Core Ideas

- Stable patterns of *E. coli* levels may be elucidated using mean relative difference (MRD) analysis.
- MRD may account for fluctuation of the level of *E. coli* in water and its relationship with water quality.
- No patterns in season nor pond layout were found to be applicable between ponds.

**Abbreviations**
AWA: agricultural water assessment
DO: dissolved oxygen
IQR: interquartile range
MPN: most probable number
MRD: mean relative difference
RFU: relative fluorescence unit
SPC: specific conductance

## 1 INTRODUCTION

### 1.1 Water as a produce contamination source

Despite industry‐wide efforts to protect the microbial safety of irrigation and other production water sources (York, 2023), fresh produce remains susceptible to contamination by bacterial pathogens. Incidence rates of foodborne illness in the United States increased in 2022 for Shiga toxin–producing *Escherichia coli*, while *Salmonella* and *Listeria* remained near their 2016–2018 levels (Delahoy et al., 2023). Produce has recently been implicated in outbreaks of foodborne illness, with irrigation water been identified as a potential reservoir for pathogens in these events (Bell et al., 2021; FDA, 2021a; Jenkins et al., 2023; Waltenburg et al., 2021).

### 1.2 Regulation of preharvest produce irrigation water

Subpart E of the Standards for the Growing, Harvesting, Packing, and Holding of Produce for Human Consumption, or Produce Safety Rule (PSR) (21 CFR Part 112.44) initially addressed the risks posed by agricultural water quality by implementing a mandatory sampling scheme and schedule for *E. coli*. However, the testing requirements were cumbersome and expensive, and foodborne pathogens were present from water sources that met the PSR standard (Antaki et al., 2016; Harris et al., 2018; Lee et al., 2018; Truitt et al., 2018; D. Weller, Love, et al., 2020). After over one decade of public input (Stone, 2024), the U.S. Food and Drug Administration revised the requirements to allow growers to qualitatively estimate the hazards within their water systems and conduct an agricultural water assessment (AWA). This assessment no longer requires *E. coli* testing, though growers may incorporate testing to guide their decision‐making as they conduct an AWA. The AWA allows for assessment based on microbial indicators, including *E. coli*, and “other relevant factors.” A need exists for research into potential monitoring tools beyond microbial testing that can evaluate a water source's suitability.

A need exists for better guidance on where to collect a water sample to test for *E. coli* to characterize the water quality of a surface water source. Guidance focuses on water quality near an irrigation system's intake (USDA SCI Division, 2022), which may change during irrigation (Stocker et al., 2020) or use terms like “representative” that are difficult to quantify (Partyka et al., n.d.). Better understanding of the patterns of *E. coli* in irrigation ponds will allow for more consistent and accurate determination of a water source's microbial quality.

### 1.3 Water use in the Georgia Coastal Plain

The coastal plain is an important growing region in the state of Georgia (Kelly, 2003), and part of the larger Southeastern Plains ecoregion (Bosch et al., 2021). Changes in water availability in the coastal plain have increased the usage of irrigation ponds, and many surface ponds are supplemented with groundwater from the Floridan aquifer, the major aquifer in this region (Hook et al., 2008; Warner, 2024; Williams & Kuniansky, 2016) to meet demand. Most irrigation ponds in the Georgia Coastal Plain are less than 25,000 m^2^ (Hook et al., 2008). These ponds are used to irrigate many economically important crops including melons, bell peppers, cucumbers, and cabbage (Georgia Farm Bureau, 2019). A mix of sprinkler and low‐flow irrigation systems are used, with 25.7% of berries, 83.1% of non‐lettuce vegetables, 26.6% of orchards, and 33.3% of lettuce and romaine irrigated with sprinklers, and the remainder irrigated with low flow methods (Haynes et al., 2023).

This study examined an array of easily measurable factors to predict *E. coli* levels in irrigation ponds. If useful, these factors may be able to be included as a critical component of an AWA, while reducing costs and labor associated with testing for generic *E. coli*.

## 2 MATERIALS AND METHODS

### 2.1 Study areas

Three ponds in the Little River watershed in Georgia (Bosch et al., 2020) were used, and information about the ponds in this study is summarized in Table 1. Ponds 2 and 3 were fed by different creeks on the same farm, the calculated watershed did not overlap. Birds, including great blue herons, cormorants, and cattle egrets, were observed at all three ponds. Feral hogs were either observed or reported at both farms. An alligator was observed in Pond 3 in October 2023. Raccoon tracks were observed occasionally at the perimeter of Pond 1. Wildlife at Ponds 2 and 3 was typically observed at the west end of the ponds, where they were fed by their respective creeks.

**TABLE 1:** Size and composition of ponds and surrounding watersheds using USDA National Agriculture Statistics Service classifications (USDA NASS, 2022).

Pond	County	Pond area (m^2^)	Average depth (m)	Watershed area (m^2^)	Watershed land cover	Land management notes
1	Worth	18,000	1.1	76,500	Non‐alfalfa hay 35.3%, 17.6% open water, 12.9% grass/pasture, 9.4% woody wetlands, 8.2% cotton, 8.2% evergreen forest, remainder peanuts, mixed forest, and wetlands	Surrounded by paddock used for cattle grazing, cotton and peanut fields with rye as a winter cover crop.
2	Tift	34,000	1.2	1,099,800	24.1% cotton, 18.3% woody wetlands, 14.2% peanuts, 12.8% evergreen forest, 10.1% non‐alfalfa hay, 6.3% corn, 6.2% developed spaces, and remainder crops and development.	Cotton and peanuts fields with fallow period.
3	Tift	43,000	1.4	1,325,700	22.5% woody wetlands, 20.4% cotton, 16.5% evergreen forest, 16.4% peanuts, 5.3% corn, 5.3% non‐alfalfa hay, and remainder non‐crop vegetation and development.	500 m north of Pond 2.

### 2.2 Sample collection

Sampling sites were evenly distributed across the ponds on a GIS map loaded into ArcGIS Field Maps (Esri) to cover various features located using a submeter‐accurate GPS (Arrow Lite, EOS Positioning Systems). Collection took place between July 2021 and September 2023. Collection typically occurred between 9:00 a.m. and 12:00 p.m. local time to reduce diurnal variation of *E. coli* levels during sampling (Stocker, Smith, et al., 2022).

**FIGURE 1:**
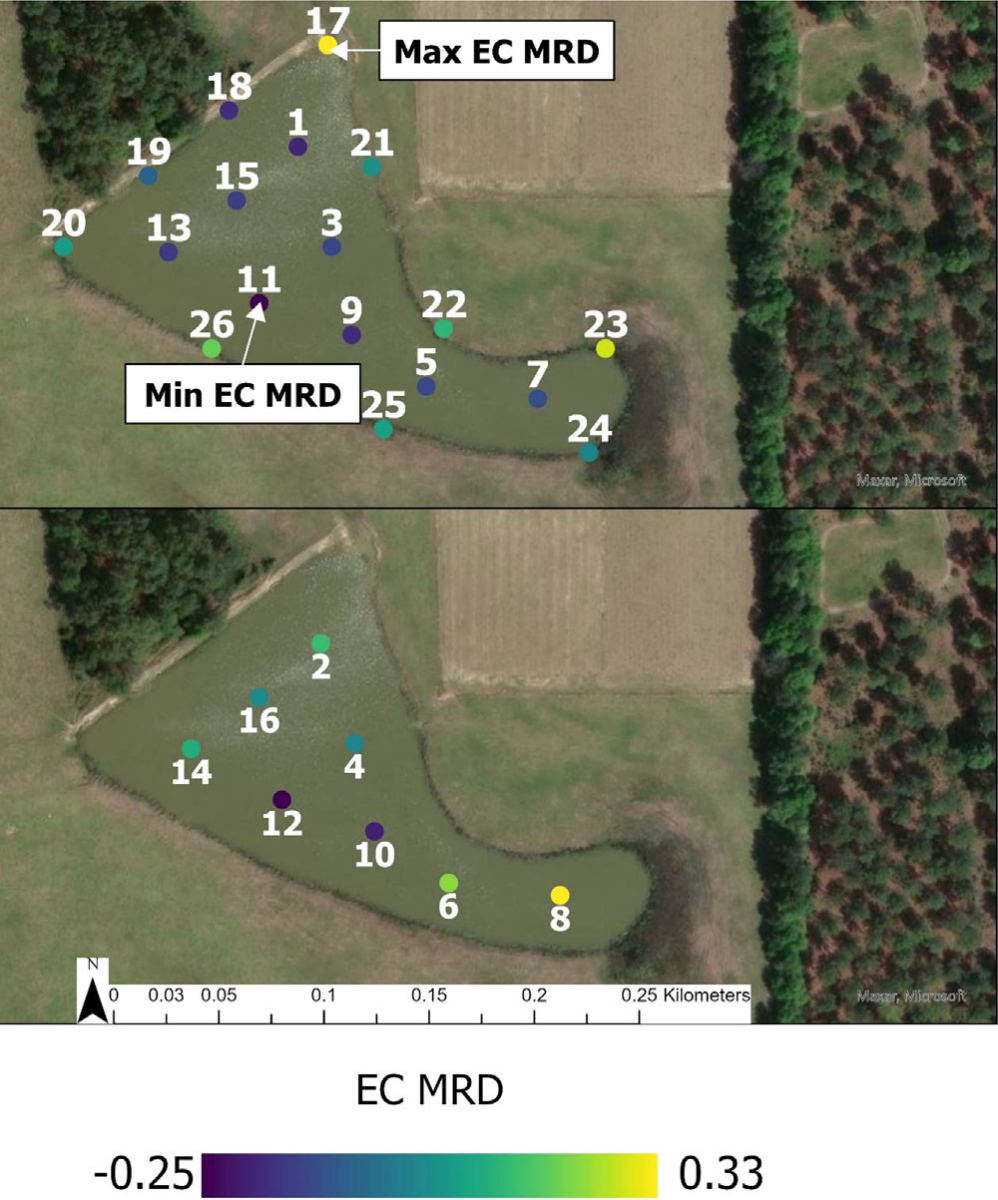
Mean relative difference (MRD) of log most probable number *E. coli* 100 mL ^−1^ distribution across locations at Pond 1 (Worth County, GA). Sampling locations are indicated by numbered dots, top picture shows samples collected from the surface or perimeter, bottom picture shows those collected from ca. 1 m subsurface. Samples were collected from July 2021 to October 2023. Collection was done twice a month, March through September, and monthly, October through April. Imagery 2024 Maxar, Microsoft.

**FIGURE 2:**
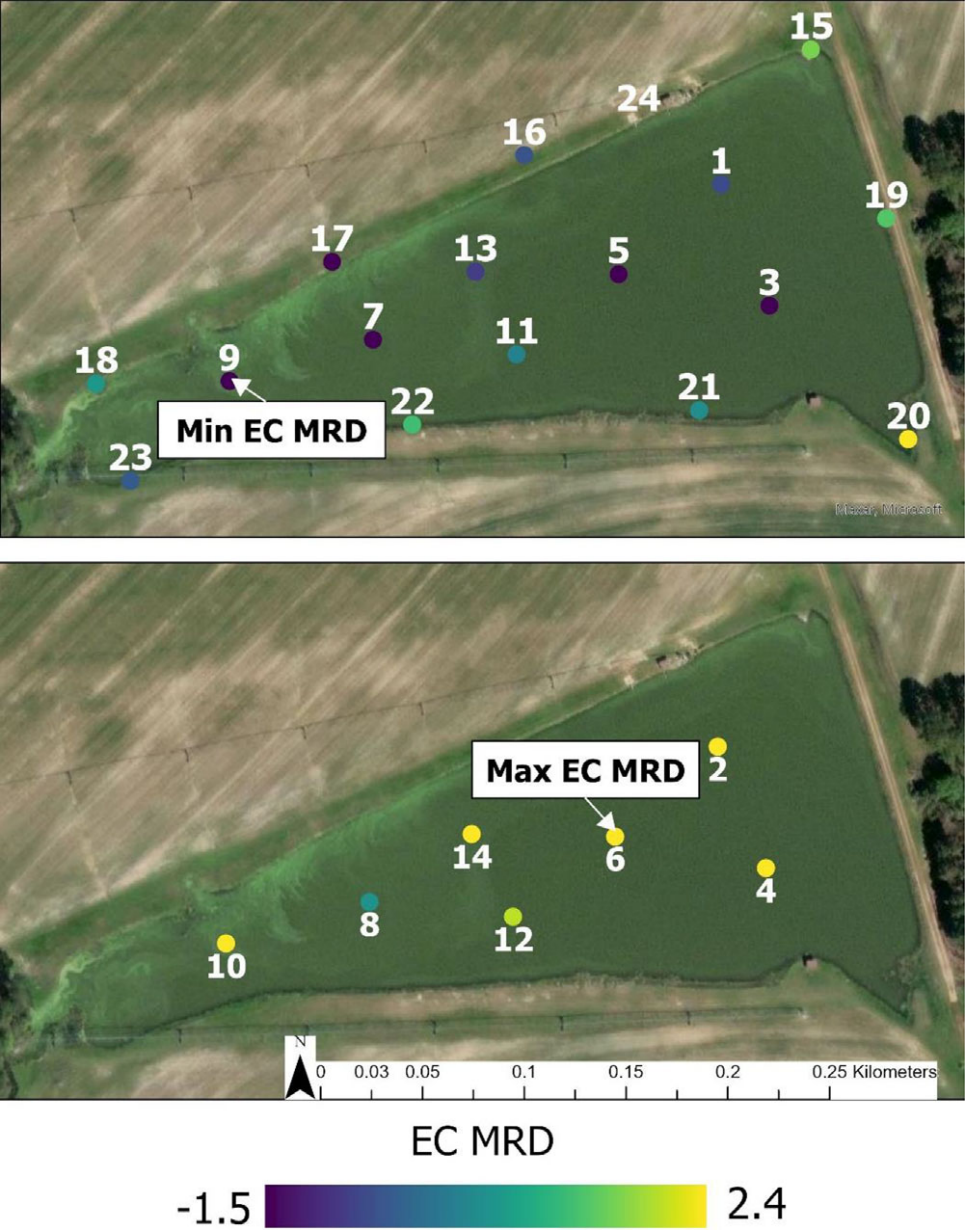
Map showing the mean relative difference of log most probable number *E. coli* 100 mL^−1^ across locations at Pond 2 (Tift County, GA). Dots are water sampling locations with corresponding sampling site number. Top picture shows samples collected from the surface and perimeter, bottom picture shows those collected from below the surface (ca. 1 m). Samples were collected from July 2021 to October 2023. Collection was done twice a month, March through September, and monthly, October through April. Imagery 2024 Maxar, Microsoft.

**FIGURE 3:**
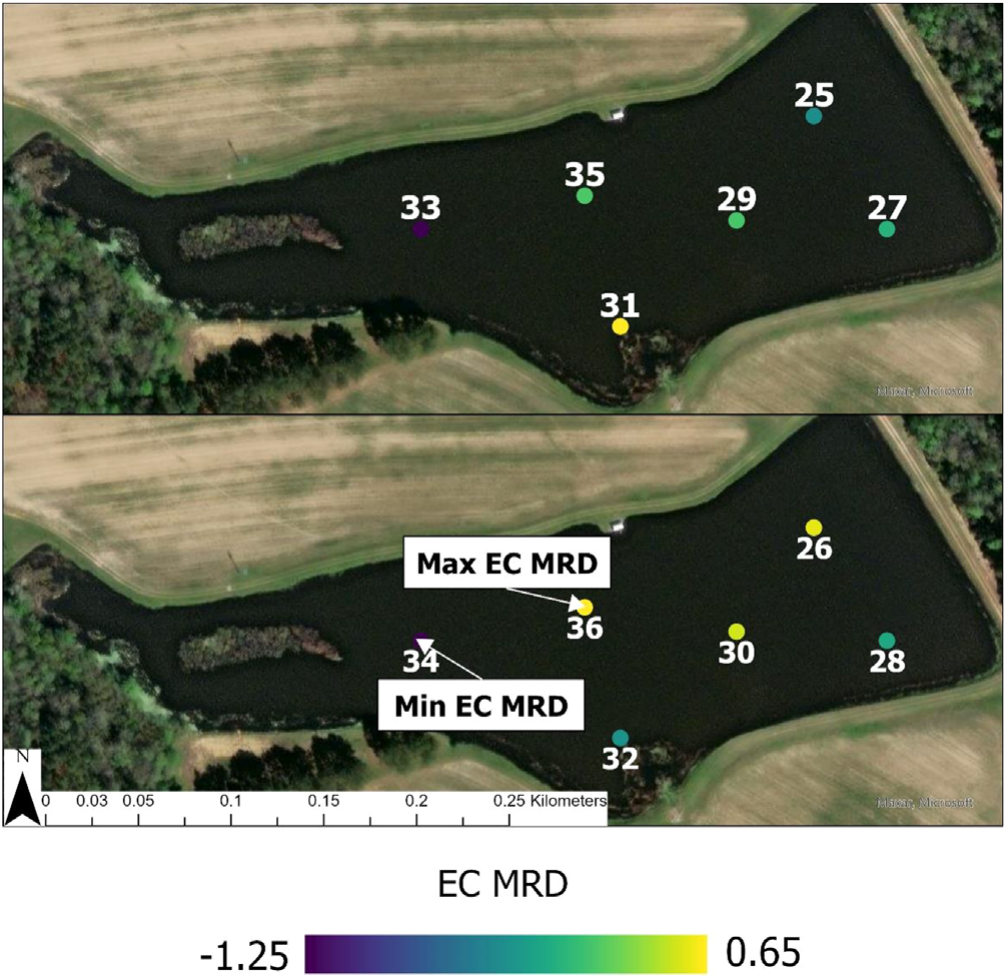
Mean relative difference of log most probable number *E. coli* 100 mL ^−1^ (MRD EC) values by location at Pond 3 (Tift County, GA). Red dots indicate where water samples were collected and are labeled with the corresponding sample number. Sample numbers in the top picture correspond to the surface samples, whereas those in the bottom figure correspond to subsurface (ca. 1 m) samples. Imagery 2024 Maxar, Microsoft.

Three types of samples were collected: surface water samples (odd samples 1–15 at Pond 1 [Figure 1], 1–13 at Pond 2 [Figure 2], and 25–35 at Pond 3 [Figure 3]), subsurface (even samples 2–16 at Pond 1, 2–14 at Pond 2, and 26–36 at Pond 3), and perimeter samples (17–26 at Pond 1, 15–23 at Pond 2, and none at Pond 3). Surface and perimeter water was collected using a 0.5‐L high‐density polyethylene sampling scoop. Depth permitting, subsurface samples were collected at 1.0 m depth for interior samples using a peristaltic pump (Sigma 900, Hach). When it was not possible to collect a sample at 1.0 m depth without sedimentation, samples were collected at 0.5 m instead. Collected water was immediately transferred into 0.5‐L sterile bottles (Nalgene) and stored in a cooler with ice packs during transport.

**Core Ideas**

- Stable patterns of *E. coli* levels may be elucidated using mean relative difference (MRD) analysis.
- MRD may account for fluctuation of the level of *E. coli* in water and its relationship with water quality.
- No patterns in season nor pond layout were found to be applicable between ponds.

After May 2022, physicochemical water quality data were collected alongside water samples using an EXO2 multiparameter sonde (YSI Inc.) with sensors for temperature (°C), pH, turbidity (nephelometric turbidity unit), dissolved oxygen (DO; mg/L), chlorophyll‐a (relative fluorescent units [RFU]), phycocyanin (RFU), fluorescent dissolved organic matter (fDOM; RFU), nitrogen‐nitrate (NO_3_‐N; mg/L), and specific conductance (SPC; µS/cm). At each interior sampling point, depth was measured with a handheld depth finder (Hondex PS‐7).

### 2.3 Enumeration of *E. coli*

Upon receipt at the lab (within 6 h of collection), sample bottles were inverted 25 times, and a 100 mL aliquot was added to a sterile glass bottle (Pyrex). Colilert reagent (IDEXX Laboratories Inc.) was combined with each sample, mixed, added to individual Quanti‐tray 2000s most probable number (MPN) trays (IDEXX Laboratories Inc.), sealed, and incubated according to manufacturer instructions. After 24‐h incubation, trays were viewed under a 365 nm UV light, fluorescent wells were marked, and *E. coli* positive wells were recorded. MPN *E. coli* 100 mL^−1^ was determined using the manufacturer's MPN table. This method is accepted by the FDA for enumeration of *E. coli* in agricultural water (FDA, 2021b).

### 2.4 Data analysis

#### 2.4.1 Data processing

Data for each pond were compiled and organized by date, then analyzed in R (R Core Team, 2022). MPN *E. coli* 100 mL^−1^ values below the limit of detection of 1.0 were replaced with 0.5, and those above the upper limit of detection of 2419.6 MPN were assigned a value of 4839.2. The MPN *E. coli* 100 mL^−1^ values were log_10_‐transformed prior to other calculations. Data manipulation was done with the “tidyverse” package (Wickham et al., 2019), and correlation visualization was done with the “corrplot” package (Wei & Simko, 2021).

#### 2.4.2 Spatial stability of *E. coli* and water quality—mean relative difference

MRD is used as a measure of temporal stability (Vachaud et al., 1985) and has been successfully applied to capture trends in bacteria, including *E. coli*, and physicochemical water quality parameters within agricultural ponds (Pachepsky et al., 2018; Kim et al., 2022; J. E. Smith et al., 2021; Stocker et al., 2021). MRD was calculated as follows: let *x_ij_* represents each measurement of variable *x* at location *i* on the *j*th sampling event, and let ${\bar{x}}_{j}\;$ represents the average across all locations during sampling *j*.

$${\bar{x}}_{j}=\frac{1}{N_{i}}\sum_{i=1}^{i=N_{i}\;}x_{ij}$$

where *N_j_* is the total number of locations. The relative differences, RD*_ij_*, for each location *i* on each sampling *j* may be calculated as follows:

$$RD_{ij}=\frac{x_{ij}-{\bar{x}}_{j}}{{\bar{x}}_{j}}$$

The MRD of *x* at location *i* is the mean value of RD*_ij_* for all sample collections *N_j_*.

$$\mathrm{MR}D_{i}=\frac{1}{N_{j}}\sum_{j=1}^{j=N_{j}\;}RD_{ij}$$

MRD values reflect the difference in measurements at a location versus the overall trend across all locations. A positive MRD indicates that *x_ij_* tends to be larger than ${\bar{x}}_{j}$, whereas a negative MRD indicates the opposite.

#### 2.4.3 Comparisons of sampling area

Data were grouped by the sampling area type: perimeter surface, interior surface, and interior subsurface (perimeter, surface, and subsurface, respectively). A Kruskal–Wallis test was used to determine a difference in median *E. coli* levels among different sampling sites followed by post hoc analysis using a pairwise Wilcoxon rank sum test using a Bonferroni adjustment with a family size of three for the areas at Ponds 1 and 2, and two for Pond 3. Nonparametric tests, medians, and interquartile ranges (IQRs) were used due to the non‐normal distribution of the MPN *E. coli* 100 mL^−1^ values even after log_10_ transformation, indicated by tailed quantile–quantile plots.

#### 2.4.4 Comparison of sampling season

Data were divided into a growing (May through September) and non‐growing (October through April) season based on produce relevant to the region. Correlation of the relative ranks of MRD was determined using Kendall's *τ* (Kendall, 1938).

#### 2.4.5 Correlation of physicochemical water quality

Correlations between physicochemical water quality measurements and log MPN *E. coli* 100 mL^−1^ were determined using Kendall's *τ* for each pond and for all observations of all ponds. Correlations were also determined only for samples of the same type: surface, subsurface, and perimeter. Correlations were performed for both the observations themselves and MRDs.

#### 2.4.6 Spatial analysis and visualization

Geospatial analysis was performed using ArcGIS Pro version 3.3.1 (Esri Inc., 2023). Watersheds were determined by importing 10 m digital elevation model data (USDA NRCS National Cartography & Geospatial Center, 1998), calculating D8 flow direction and accumulation, creating a pour point at each pond's outflow then using the watershed tool. Watershed composition came from the National Agricultural Statistic Service's 30 m cropland dataset (USDA NASS, 2022).

## 3 RESULTS

### 3.1 Differences by sampling area

The MPN *E. coli* 100 mL^−1^ differed significantly based on sampling area and sampling location for all three ponds according to a Kruskal–Wallis test (Table 2). Pairwise Wilcoxon comparison of Pond 1 found that all sampling areas significantly differed from one another. At Pond 2, all areas significantly differed, except for perimeter and subsurface samples collected at the same location. Pond 3's surface and subsurface samples significantly differed.

**TABLE 2:** Comparison of median log MPN 100 mL^−1^ across pond and sampling area.

	Median log MPN 100 mL^−1^ (interquartile range)		
Area	Pond 1 (*n* = 936)	Pond 2 (*n* = 829)	Pond 3 (*n* = 432)
Perimeter	1.80^*^ (1.05)	0.80^*^ (1.4)	–
Surface	1.63^*^ (0.72)	0.30 (0.96)	0.49^*^ (1.2)
Subsurface	1.49^*^ (0.72)	0.30 (1.34)	0.72^*^ (1.4)

* A significant difference (*p* < 0.05) within each pond according to a Kruskal–Wallis test followed by a pairwise Wilcoxon comparison of the medians of each area.

When grouped together as surface, subsurface, or perimeter samples, a Kruskal–Wallis test comparing the *E. coli* levels by location was only significant for subsurface samples at Pond 3 (*χ* ^2^ = 16.097, *df* = 5, *p* = 0.02 after a Bonferroni adjustment for three tests).

**FIGURE 4:**
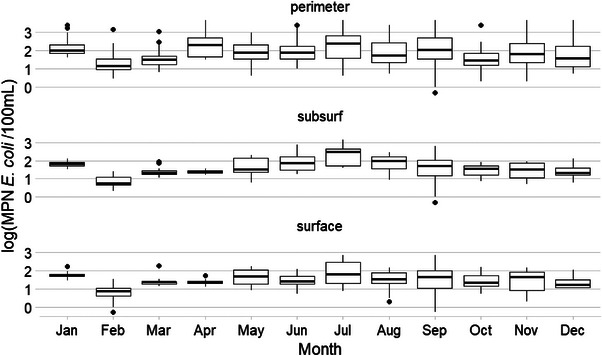
Boxplot of *E. coli* levels across area by sampling month at Pond 1.

The highest median *E. coli* level at Pond 1 (Figure 4; *n* = 936) was from subsurface samples collected in July, and the lowest was from subsurface samples collected in February. The highest IQR of 1.26 was observed in perimeter samples collected in July, and the lowest was 0.13 in April subsurface samples. Maximum *E. coli* MRD (EC MRD) was 0.33 at Point 17, and minimum was −0.25 at Point 11. EC MRD tended to be higher for subsurface samples and the north and eastern points of the pond (Figure 1).

**FIGURE 5:**
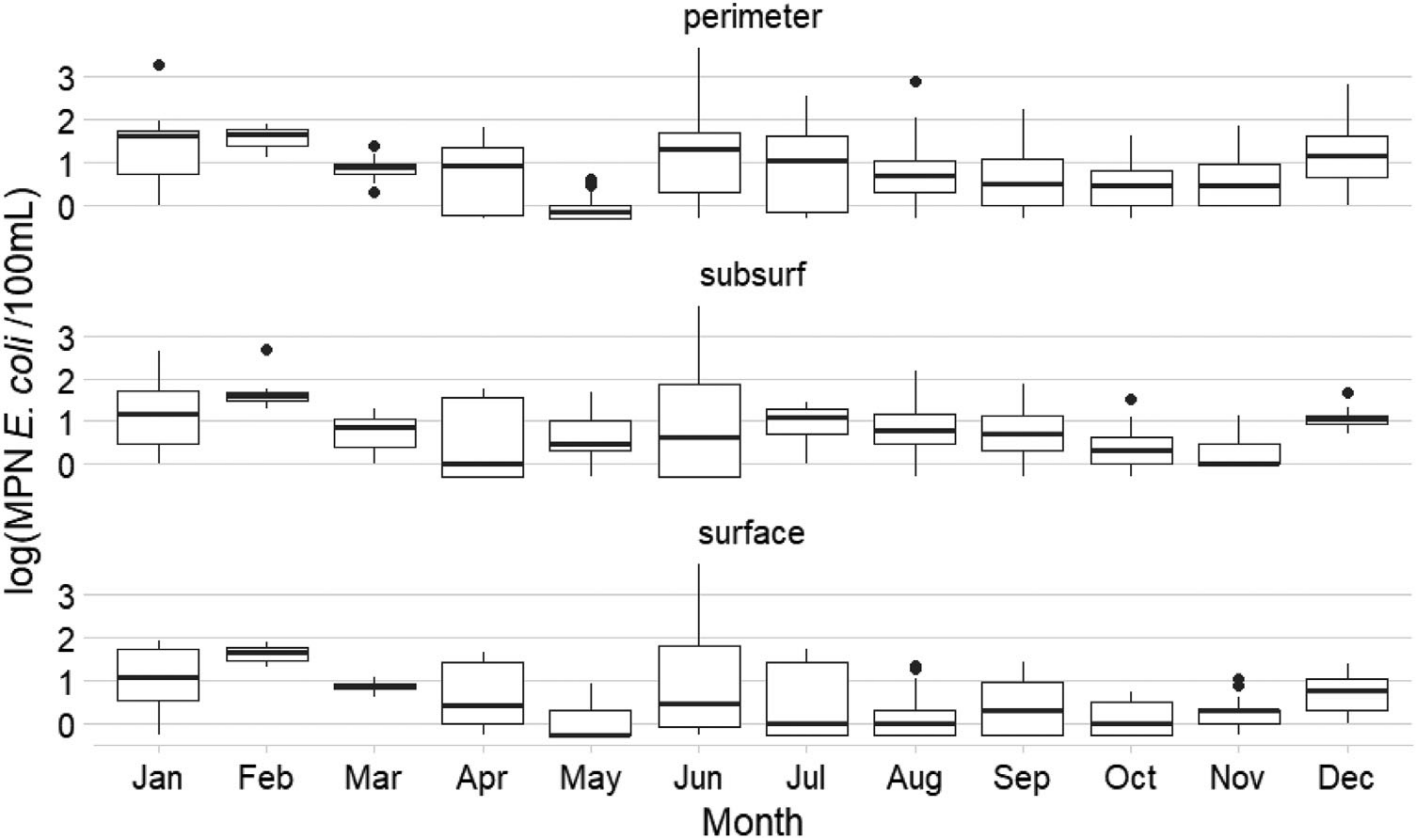
Boxplot of *E. coli* levels at Pond 2 by sampling area and month.

Pond 2 (Figure 5; *n* = 829) had its highest median *E. coli* levels in perimeter samples collected in February, and the lowest from surface samples collected in May. June subsurface samples had the greatest IQR of 2.15 and March surface samples had the lowest IRQ of 0.13. Location 6 had the maximum EC MRD of 2.4, and Location 9 had the minimum of −1.5. EC MRD, which tended to be higher for subsurface and perimeter samples on the western and eastern ends of the ponds (Figure 2).

**FIGURE 6:**
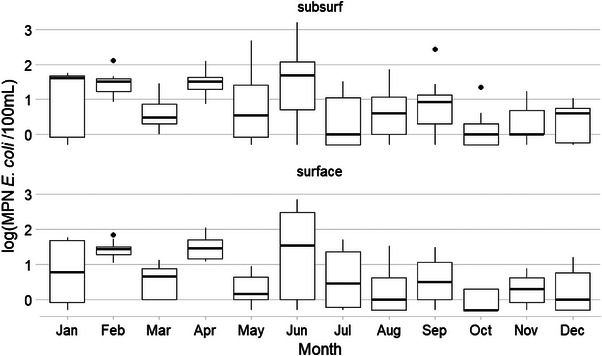
Boxplot of *E. coli* levels at Pond 3 by sampling area and month.

At Pond 3 (Figure 6; *n* = 432), June surface samples had the highest IQR of 2.48 compared to 0.21 in February surface samples. Location 36 had the maximum EC MRD of 0.65, and location 34 had the lowest EC MRD of −1.25. EC MRD values were lower on the western end of the pond and higher among subsurface samples compared to surface, except for the shallow end on the south side (Points 31/32) of the pond, where this pattern reversed (Figure 3).

### 3.2 Relationship between water depth and EC MRD

There was no significant correlation between the EC MRD of the surface or subsurface samples at a location (i.e., corresponding surface and subsurface locations did not follow the same trends), nor was there a significant correlation between EC MRD of surface or subsurface samples with average water depth. The difference in surface and subsurface EC MRD at a given location was not significantly correlated with water depth at any location (i.e., collecting from a subsurface location in shallow water did not mean that it more closely followed its corresponding surface location).

Difference in each measured depth from average depth was determined at each location. The MPN *E. coli* 100 mL^−1^, SPC, NO_3_‐N, and temperature were correlated with difference in depth. For surface samples, there was no significant correlation (*p* > 0.05) at Ponds 1 and 2, but Pond 3 had a weak positive (*τ* = 0.27, *p* < 0.001) correlation between level of *E. coli* and difference in depth (i.e., as the water level was higher, there were typically higher *E. coli* levels) and a moderate negative (*τ* = −0.43, *p* < 0.001) correlation between SPC and depth difference. For subsurface samples, there was no significant correlation at the first two ponds, but Pond 3 had a weak positive correlation between *E. coli* level (*τ* = 0.19, *p* = 0.001), and weak negative correlation for temperature (*τ* = −0.25, *p* = 0.006) and depth difference.

### 3.3 Correlation of *E. coli* with physicochemical water quality

**FIGURE 7:**
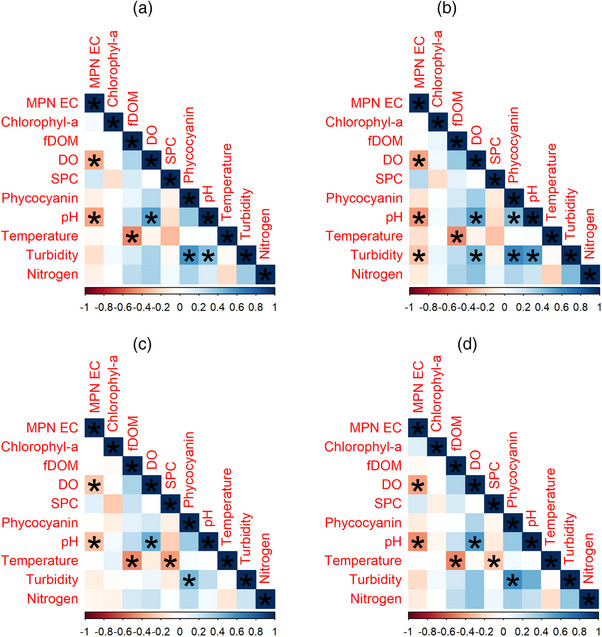
Pond 2 Kendall's correlation plots, a = overall, b = surface, c = subsurface, and d = perimeter. *Significance at a level of *p* < 0.05.

At Pond 1 (*n* = 563) and Pond 3 (*n* = 198), there were no significant correlations (*p* > 0.05) between any of the physicochemical parameters measured and the levels of *E. coli* as determined by Kendall's *τ*. At Pond 2 (Figure 7; *n* = 476), the level of *E. coli* for all samples correlated negatively with dissolved oxygen (*τ* = −0.36, *p* = 0.005) and positively with pH (*τ* = 0.40, *p* = 0.002). For surface samples (*n* = 142), dissolved oxygen (*τ* = −0.40, *p* = 0.005), pH (*τ* = −0.42, *p* = 0.002), and turbidity (*τ* = −0.28, *p* = 0.02) were significantly correlated with *E. coli*. Subsurface (*n* = 136) and perimeter (*n* = 198) samples had a significant negative correlation of *E. coli* levels with dissolved oxygen (*τ* = −0.27, *p* = 0.03; *τ* = −0.40, *p* = 0.001) and pH (*τ* = −0.29, *p* = 0.02; *τ* = = 0.45, *p* = 0.0005).

**FIGURE 8:**
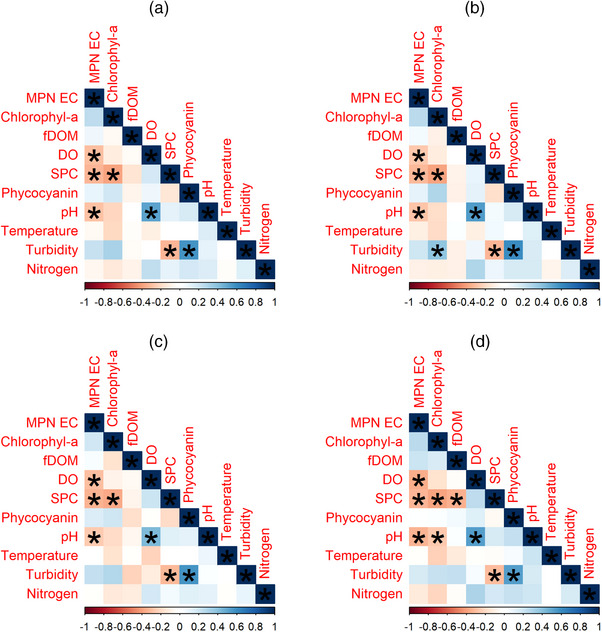
Overall dataset correlation plots, a = all areas, b = surface, c = subsurface, and d = perimeter.

The combined dataset, which pooled observations from all three ponds (Figure 8; *n* = 1237), had significant negative correlations between the level of *E. coli* and dissolved oxygen (*τ* = −0.30, *p* = 0.02), SPC (*τ* = −0.33, *p* = 0.03), and pH (*τ* = −0.29, *p* = 0.02). These same quality measures were significantly negatively correlated with *E. coli* levels across all three sampling areas: surface (DO *τ* = −0.27, *p* = 0.046; SPC *τ* = −0.32, *p* = 0.045; pH *τ* = −0.27, *p* = 0.036), subsurface (DO *τ* = −0.30, *p* = 0.03; SPC *τ* = −0.33, *p* = 0.029; pH *τ* = −0.25, *p* = 0.04), and perimeter (DO *τ* = −0.36, *p* = 0.004; SPC *τ* = −0.33, *p* = 0.026; pH *τ* = −0.34, *p* = 0.008).

### 3.4 Correlation of EC MRD values with physicochemical water quality

**FIGURE 9:**
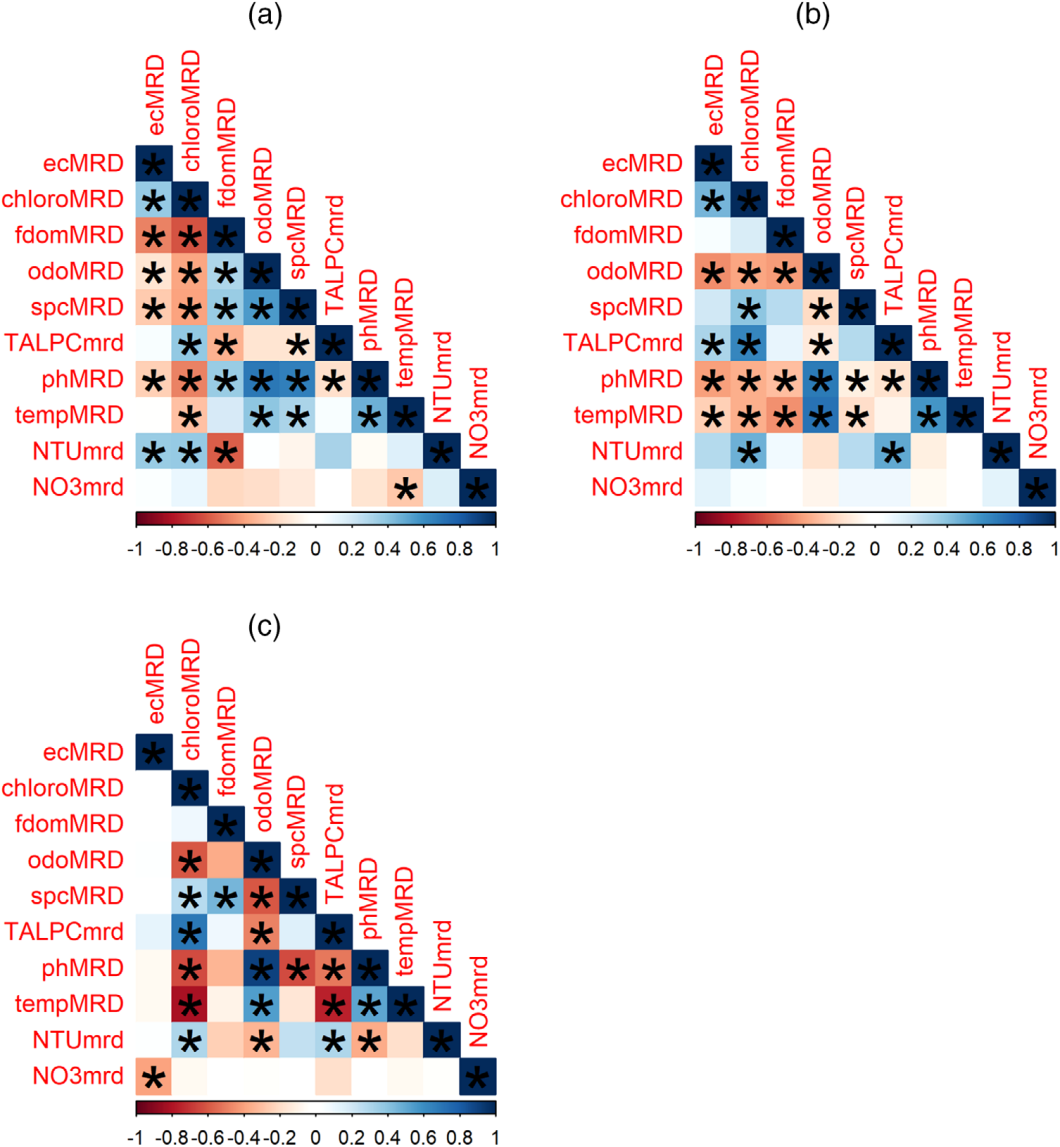
Correlation plots for MRD values of each pond, a = Pond 1, b = Pond 2, and c = Pond 3.

All ponds had significant correlations between their EC MRD values and at least one of the physicochemical parameters' MRD values (Figure 9). At Pond 2, the MRDs of chlorophyll‐a (*τ* = 0.45, *p* = 0.006) and phycocyanin (*τ* = 0.31, *p* = 0.04) correlated positively with EC MRDs, whereas those of DO (*τ* = −0.45, *p* = 0.002), pH (*τ* = −0.41, *p* = 0.001), and temperature (*τ* = −0.25, *p* < 0.001) had significant negative correlations. Only the NO_3_‐N MRD values had a significant negative correlation (*τ* = −0.39, *p* = 0.02) at Pond 3. Pond 1 had significant positive correlations between EC MRDs and those of chlorophyll‐a (*τ* = 0.34, *p* = 0.012) and turbidity (*τ* = 0.366, *p* = 0.02) and negative with fDOM (*τ* = −0.48, *p* = 0.003), DO (*τ* = −0.17, *p* = 0.05), SPC (*τ* = −0.27, *p* = 0.02), and pH (*τ* = −0.24, *p* = 0.03).

## 4 DISCUSSION

### 4.1 Seasonal differences in distribution of *E. coli*

There was no significant difference (*p* > 0.05) in the rank correlation of EC MRD values between harvest and non‐harvest seasons, or overall EC MRD values at any of the three ponds. These findings indicate that focusing sampling efforts only during the harvest season is suitable.

No pattern in months featuring higher *E. coli* levels was observed. For example, January and February saw the highest median *E. coli* levels at Pond 2 (Figure 5), whereas February and December had the lowest *E. coli* levels at Pond 1 (Figure 4). Pond 3 (Figure 6) had its highest *E. coli* levels in June, but second highest in January. This may indicate that a difference in the management of the landscape around the ponds, such as the use of cover crops at Pond 1 during the winter or the cattle entering the pond less frequently in the colder winter months, may have led to lower *E. coli* levels in the pond (Blanco‐Canqui, 2018).

### 4.2 Differences by sampling area

#### 4.2.1 Variability of *E. coli* levels

*E. coli* levels can range drastically within the same pond during the same month, as seen with June surface samples at Pond 3 (Figure 6). Previous work on *E. coli* in ponds found that spatial factors were more responsible for variance than other factors, such as the sampling date or physicochemical measurements (Murphy et al., 2023). The same study also found that in as little as 57 m of distance along the shoreline, measurements of fecal indicator bacteria, including *E. coli*, became statistically independent from neighboring points. In our study, the average distance between sampling points at Pond 1 was 46 m for interior sampling points and 79 m for perimeter points, 63 and 102 m at Pond 2, and 79 m at Pond 3. Only Pond 3's subsurface samples differed significantly from one another despite the space between points.

Repeated sampling of the same location, when chosen to represent the overall pond's microbial water quality (i.e., an MRD value close to 0) should reduce the variability of the *E. coli* measurements. However, this work did not identify specific features that could identify locations with a representative MRD or an extreme MRD. Perimeter points such as 19, 21, and 24 at Pond 1 (Figure 1) or 15, 18, 19, and 22 at Pond 2 (Figure 2) provided good estimates of the overall water quality. Perimeter sampling may be appropriate for estimating the *E. coli* content of a body of water, though collecting at multiple points may ensure one does not select points that deviates from the overall water quality (e.g., Points 17 or 23 at Pond 1, Figure 1; Point 20 at Pond 2, Figure 2).

### 4.3 Spatial patterns of *E. coli*

Previous work in this region examined large volumes of water at a limited number of locations, typically near the irrigation pump (Antaki et al., 2016; G. Gu et al., 2013). The current study, however, sampled multiple locations from the same pond on the same sampling day. For example, at Pond 1, on a day with a mean log MPN *E. coli* 100 mL ^−1^ of 1.70 (the mean of all samples at this pond), Point 17's EC MRD of 0.33 and Point 11's MRD of −0.25 would imply log MPN *E. coli* levels of 2.26 and 1.28, a difference of 0.98.

Focusing sampling efforts near the pump intake has been used as an ideal measure of the water quality that will be delivered into the distribution system (Antaki et al., 2016; Lee et al., 2018). Points 2 and 4, closest to the two pivot intakes at Pond 2 have high EC MRD values (1.6 and 2.3, respectively), and *E. coli* levels from samples taken elsewhere in the pond may not accurately reflect the area immediately around the intake. Point 36 at Pond 3, closest to its pivot's intake, had the highest EC MRD, 0.65, for that pond. Pond 1 was not being used for irrigation during the study, intake would be near Point 4, with an EC MRD of 0.04, which closely aligned with the overall level of *E. coli* in the pond. Water quality at the intake is also impacted, with a simulated irrigation event showing a change in *E. coli* levels over time at the pump intake of an irrigation pond (Stocker et al., 2020).

The overall water quality of the pond needs to be considered, not just near the intake due to the volume of water used in irrigation. Pond 2's south pivot irrigates 275,000 m^2^. At a rate of 0.6 in./acre (ca. 15 mm/4047 m^2^) (McAvoy & Coolong, 2017) for leafy greens in the sandy soils found in Tift County, 3.4 acre‐ft (4194 m^3^) of water would be drawn from the pond, reducing the depth by 0.4 ft (0.12 m), drawing water from throughout the pond.

### 4.4 Differences by sampling area

At Ponds 1 and 2, all surface samples had EC MRD values below 0, while Pond 3's second highest EC MRD was found at a surface location. Pond 2's three highest EC MRD values were subsurface samples, whereas at Pond 1 the three highest MRD values were perimeter samples. Sampling only the perimeter at Pond 2 would underestimate the *E. coli* levels of the water being taken up by the pivot's pump, whereas at Pond 1, these measurements would be an overestimate. This highlights the utility in collecting many samples at the beginning of a monitoring program, which allows areas with extremes in concentrations to be identified and excluded (Stocker et al., 2021). Water quality managers in this region may also benefit from collecting samples from different parts of the water column to better understand any bias that may arise from sampling depth.

#### 4.4.1 Influence of water depth

The correlation between average depth and *E. coli* levels observed only at Pond 3 suggests that water level may be relevant at some ponds. Pond 3 was the deepest and largest pond surveyed and was fed by the largest watershed. Pond 3 is also nearly twice the size of the average pond used for irrigation in Georgia (Hook et al., 2008), so this relationship may not be representative of more typical ponds in the region. Influence of depth may also be affected by the amount of mixing, which is most significant in the top meter of water (Henderson et al., 2024)

### 4.5 Pond design as a potential preventive measure

No relationship was found between the depth of the ponds and the levels of *E. coli*, so changes in the depth of a pond would not be an effective strategy to manage water quality. Effective management of runoff and erosion may help reduce *E. coli* levels at the perimeter of agricultural ponds (Hawkins & Wallace, 2013). To reduce contamination from animal agriculture, animals and other foreseeable sources of hazards should be excluded from the growing area and water sources [21 CFR 112.83(b) and (21 CFR 112.42)]., and runoff controls such as conservative tillage or vegetative buffers may be used. Natural biofiltration and vegetation has also been associated with lower levels of *E. coli* in irrigation waters (D. L. Weller et al., 2022).

#### 4.5.1 Correlations of water quality with levels of *E. coli*

Significant correlations between the measured physicochemical water quality parameters and the MPN *E. coli* were found for Pond 2 and the combined ponds datasets, but not independently for the other two ponds. While DO and pH were significant covariates across all areas and the overall water quality for Pond 2, only the surface samples had turbidity as a significant covariate as well. The lack of correlation observed at Pond 3, located a few hundred meters north of Pond 2 on the same farm, highlights the difficulty in translating patterns seen in one water body to another. Variability between the two may be due to differences in size and average depth, the differences in watershed size and inputs, the use of groundwater to supplement the south pond, differences in aquatic vegetation, or any combination of these.

All correlations observed were weak to moderate (Gilpin, 1993; Schober et al., 2018) and patterns may be better elucidated with modeling. Modeling efforts may have to account for differences by area and pond, which this work showed to be significant. Water quality datasets such as the one generated by this study are often unbalanced, this must be accounted for when creating training and test datasets, which may be done with synthetic minority oversampling (D. L. Weller et al., 2021a) or quantile‐based splitting (Hong et al., 2024). It is promising that the covariates that were significant at Pond 2 and for the dataset composed of all ponds' data (DO, SPC, pH, and turbidity) may be practicably, rapidly, and economically measurable in the field or continuously in a distribution system. If these correlations are found to be useful indicators of a hazard in conjunction with or in lieu of *E. coli* levels, it may be feasible to incorporate them into a monitoring regime for surface water sources.

#### 4.5.2 Correlation of physicochemical MRD values with EC MRD

Compared to the correlations with *E. coli* levels that were determined on a per‐sample basis, there were many more significant correlations between the MRD values of covariates and EC MRD. Particularly of note are the negative correlations seen between the levels of phycocyanin at Pond 2 (Figure 9a) and positive correlation with chlorophyll‐a levels at Pond 1 (Figure 9c). These trends may suggest that *E. coli* distribution at Pond 2 is limited by the presence of cyanobacteria (which produce phycocyanin), while the distribution is associated with microalgae (which use chlorophyll) at Pond 1. This is consistent with previous research, which found that *E. coli* utilizes the carbon produced by microalgae in carbon‐poor conditions (Cho et al., 2022). It was reported that *E. coli* in wastewater treatment systems utilize the carbon released by green algae (Bouteleux et al., 2005). Meanwhile, *E. coli* levels decreased in the presence of *Mircocystis* species (a cyanobacteria) (Zhou et al., 2023). The negative correlation between nitrogen and EC MRD at Pond 3 may be due to the deleterious effect that exogenous *E. coli* has on nitrification (L. Gu et al., 2021), though this would require further investigation. Water quality measurements, including DO, pH, and SPC, also provide utility in monitoring for harmful algal blooms in freshwater systems (Ahn et al., 2021; S. A. Smith, 2021), which may reduce water quality and clog water distribution systems. Algal blooms are typically associated with a decrease in DO and an increase in pH and conductivity (YSI Inc., n.d.).

Analysis of datasets gathered from streams and canals (D. L. Weller et al., 2021b) in New York and Arizona (D. Weller, Belias, et al., 2020; D. Weller, Brassill, et al., 2020) had turbidity and DO as the fourth and ninth highest permutation‐based variables for *E. coli* level, respectively. D. L. Weller et al. (2021b) found average temperature 5–10 days and 10–20 days before sampling and rain 0–1 days before sampling were more important predictors than turbidity. A study on irrigation ponds in Maryland used a similar spatial design to this project (Stocker, Pachepsky et al., 2022) and found that SPC, turbidity, temperature, chlorophyll, and fDOM were the most important predictors of *E. coli* levels, whereas at separate study sites those same authors reported that fDOM, turbidity, and phycocyanin correlated well with *E. coli* levels (Stocker et al., 2019). The relationships between environmental covariates and *E. coli* are largely site‐specific, though some covariates are more consistent across studies. Future work is needed to better generalize these associations in surface irrigation water sources. Because correlations of MRDs provided stronger results than the pointwise analysis, future work may focus on using stable patterns of covariates as predictors of indicator or pathogen densities.

### 4.6 Study limitations

This study focused only on *E. coli* as measured by the IDEXX Quanti‐tray 2000 with Colilert. While this method has been accepted as equivalent to previous filtration‐based methods for enumerating *E. coli* in water (Buckalew et al., 2006; FDA, 2021b), various bacteria and algae may have the potential to cause false‐positives for the 4‐methylumbelliferon product responsible for the fluorescence of the wells (Davies et al., 1994; Pisciotta et al., 2002), these studies used marine water and species instead of freshwater. The farms used in this study did not grow fresh produce, and practices used when growing commodities may affect water quality differently than those on produce farms. This study focused on stable patterns of *E. coli* and water quality and did not examine the effects that weather, particularly precipitation, solar irradiance, or wind, might have on individual measurements of *E. coli*.

## 5 CONCLUSION

It is difficult to determine surface water quality. Differences in seasonal patterns, overall *E. coli* levels, and factors associated with those levels reinforce the need to individually assess each water source. Sampling without consistency in location or sample type may not accurately represent the overall level of *E. coli* at a pond on that sampling day. Spatial patterns of *E. coli* within each pond, determined using MRD, suggest that sampling from the middle of the bank at multiple locations may provide a more representative estimate of *E. coli* levels across the pond. MRD analysis may be useful for water quality managers as a method of accounting for the spatial variability of *E. coli*, helping to ensure that higher observed *E. coli* levels are due to increased contamination instead of sampling from a different area. Environmental covariates may provide instantly measurable indications of pond conditions and help determine if additional testing or action is needed, though relationships may be specific to a pond. Additional analysis will be needed to see if these patterns found in a fecal indicator are applicable to enteric foodborne pathogens found in agricultural water and the possibility of modeling and predicting their occurrence.

## AUTHOR CONTRIBUTIONS

**J. Andrew Widmer**: Data curation; formal analysis; investigation; writing—original draft. **Matthew Stocker**: Conceptualization; formal analysis; methodology; resources; writing—review and editing. **Jaclyn E. Smith**: Conceptualization; investigation; writing—review and editing. **Alisa Coffin**: Conceptualization; resources. **Oliva Pisani**: Resources; writing—review and editing. **Timothy Strickland**: Conceptualization; project administration; resources; writing—review and editing. **Manan Sharma**: Conceptualization; methodology; project administration; supervision; writing—review and editing. **Yakov Pachepsky**: Conceptualization; data curation; investigation; methodology; project administration; resources; supervision; visualization; writing—review and editing. **Laurel L. Dunn**: Conceptualization; resources; supervision; writing—review and editing.

## CONFLICT OF INTEREST STATEMENT

The authors declare no conflicts of interest.
